# Glucosylceramide Plays a Role in Fungal Germination, Lipid Raft Organization and Biofilm Adhesion of the Pathogenic Fungus *Scedosporium aurantiacum*

**DOI:** 10.3390/jof6040345

**Published:** 2020-12-08

**Authors:** Victor Pereira Rochetti, Rodrigo Rollin-Pinheiro, Evely Bertulino de Oliveira, Mariana Ingrid Dutra da Silva Xisto, Eliana Barreto-Bergter

**Affiliations:** Laboratório de Química Biológica de Microrganismos, Departamento de Microbiologia Geral, Instituto de Microbiologia Paulo de Góes, Universidade Federal do Rio de Janeiro (UFRJ), Rio de Janeiro 21941-902, Brazil; victorrochetti@gmail.com (V.P.R.); rodrigorollin@gmail.com (R.R.-P.); evelyoliveira4@outlook.com (E.B.d.O.); marylanax@gmail.com (M.I.D.d.S.X.)

**Keywords:** *Scedosporium*, glucosylceramide, fungal growth, biofilm

## Abstract

Infections caused by *Scedosporium* species present a wide range of clinical manifestations, from superficial to disseminated, especially in immunocompromised patients. Glucosylceramides (GlcCer) are glycosphingolipids found on the fungal cell surface and play an important role in growth and pathogenicity processes in different fungi. The present study aimed to evaluate the structure of GlcCer and its role during growth in two *S. aurantiacum* isolates. Purified GlcCer from both isolates were obtained and its chemical structure identified by mass spectrometry. Using ELISA and immunofluorescence techniques it was observed that germination and NaOH-treatment of conidia favor GlcCer exposure. Monoclonal anti-GlcCer antibody reduced germination when cultivated with the inhibitor of melanin synthesis tricyclazole and also reduced germ tube length of conidia, both cultivated or not with tricyclazole. It was also demonstrated that anti-GlcCer altered lipid rafts organization, as shown by using the fluorescent stain filipin, but did not affect the susceptibility of the cell surface to damaging agents. Anti-GlcCer reduced total biomass and viability in biofilms formed on polystyrene plates. In the presence of anti-GlcCer, germinated *S. aurantiacum* conidia and biofilms could not adhere to polystyrene with the same efficacy as control cells. These results highlight the relevance of GlcCer in growth processes of *S. aurantiacum*.

## 1. Introduction

*Scedosporium* and *Lomentospora* species are ubiquitous filamentous fungi, commonly found in areas impacted by humans. They cause disease in both immunocompetent and immunocompromised patients, ranging from localized to disseminated infections [1,2,3]. These fungal species are multidrug resistant and also highly associated with respiratory tract colonization in cystic fibrosis patients [4,5,6]. Within the *Scedosporium* genus, *Scedosporium aurantiacum* is a clinically relevant species that was related to severe disseminated infections and brain abscesses [7,8]. In experimental models *S. aurantiacum* was demonstrated to be as virulent as *Lomentospora prolificans* and relatively more virulent than other *Scedosporium* species [9,10]. Some aspects correlated to *S. aurantiacum* virulence include the capacity for germination under host conditions and rapid formation of robust biofilms on various types of surfaces, such as central venous catheter and tissue culture dishes [11,12].

The fungal cell wall is an important structure that possesses a variety of glycoconjugates that are related to biological properties and pathogenesis of the fungus [13]. Glucosylceramides (GlcCer) are the main neutral glycosphingolipids found on the cell surface of the majority of fungi and consist of a single glucose residue in glycosidic linkage to the C-1 hydroxyl group of a lipid moiety called ceramide. GlcCer structures are conserved among fungi and present a C8-desaturated and C9-methylated sphingoid base which is not found in GlcCer from mammalian cells [14,15]. The specific fungal GlcCer structure was shown to be relevant for biological properties such as virulence [16,17]. GlcCer structures isolated from *Scedosporium* and *Lomentospora* species are composed of a glucose unit with fatty acid chains varying in length and degree of saturation [18,19,20].

GlcCer molecules are associated with growth, yeast-to-hyphae transition, hyphal elongation and virulence in major fungal pathogens, such as *Cryptococcus neoformans*, *Candida albicans* and *Aspergillus nidulans* [16,21,22,23]. It has already been demonstrated that the glycosphingolipid biosynthetic pathway is crucial for biofilm formation in *S. boydii* and *A. fumigatus* [24,25]. In this context, monoclonal antibodies binding to fungal GlcCer (anti-GlcCer) are a useful tool for studying their biological functions. Anti-GlcCer protect mice against *C. neoformans* infection, decrease *Colletotrichum gloeosporioides* germination, present synergistic effect with itraconazole against *S. apiospermum* and enhance phagocytosis and microbicidal activities by macrophages in *S. apiospermum*, *S. aurantiacum* and *S. minustisporum* [19,26,27,28].

Since fungal GlcCer structures are different from mammalian GlcCer and are associated with fungal physiology and virulence in different *Scedosporium* species, studies on structure and function of GlcCer become important as they could be considered potential targets for new therapeutic strategies. In this work, we aimed to characterize the chemical structure of GlcCer of different isolates of *S. aurantiacum*, and to use monoclonal anti-GlcCer antibodies to analyze the cell surface distribution and the relevance of these molecules in growth processes, including hyphal elongation and biofilm formation.

## 2. Materials and Methods

### 2.1. Microorganisms and Growth Conditions

*Scedosporium aurantiacum* WM 09.12, an environmental strain isolated from a soil sample, and *Scedosporium aurantiacum* WM 06.385, a clinical strain isolated from a patient with a colonized ear were kindly provided by Wieland Meyer from the Sydney Institute of Infectious Diseases and Biosecurity, The University of Sydney. Both *S. aurantiacum* strains were maintained in liquid Sabouraud medium (0.5% yeast extract, 1% peptone, and 2% glucose). Conidia were obtained by scraping with PBS the surface of 7-days grown cultures (at room temperature) of Sabouraud-agar plates. To remove hyphal fragments and debris, the collected conidia were filtered and washed twice with sterile PBS. Tricyclazole (DHN-melanin synthesis inhibitor, Sigma-Aldrich, MO, USA) was added at 16 µg/mL in Sabouraud-agar plates to obtain non-melanized conidia [29].

### 2.2. Extraction and Purification of S. aurantiacum GlcCer

200 g (wet weight) of hyphae and conidia from both *S. aurantiacum* isolates were treated with chloroform/methanol 2:1 and 1:2 (*v*/*v*) for total lipid extraction. The crude lipid extracts were partitioned as described by Folch et al. (1957) and the lower layer was fractionated on a silica gel column, according to Calixto et al. [18]. Fractions were analyzed by thin layer chromatography and those containing purified GlcCer were collected and used for chemical analysis.

### 2.3. Sugar Analysis

GlcCer was hydrolyzed with 3 M trifluoroacetic acid (TFA) at 100 °C for 3 h and the resulting monosaccharides were analyzed by high performance thin layer chromatography according to Rollin-Pinheiro et al. [28].

### 2.4. ESI-MS Analysis of S. aurantiacum GlcCer

Analysis of purified GlcCer was performed by electrospray ionization mass spectrometry (ESI-MS) in positive mode using an ESI-ion Trap instrument (Model Amazon SL, Bruker, Germany). Nitrogen was used as a nebulizer and carrier gas. Samples were processed according to Calixto et al. [18]

### 2.5. Reactivity of Anti-GlcCer with S. aurantiacum Conidia, Germinated Conidia and Mycelia

The reactivity of the anti-GlcCer antibody was evaluated by ELISA as described by Xisto et al. [20] with some modifications. Anti-GlcCer antibody was previously obtained according to Rollin-Pinheiro et al. [28] Flat-bottomed polystyrene microtiter plates were coated with 10^6^ conidia/mL grown in RPMI (Roswell Park Memorial Institute) 1640 for 0 (non-germinated conidia), 3, 6 and 18 h at 37 °C. Conidia treated with 1 M NaOH overnight (for a partial depletion of melanin) and untreated conidia were also used. All fungal cells were fixed in 4% paraformaldehyde in order to obtain different stages of fungal growth. Plates were washed three times with PBS and blocked with PBS-BSA (1%). Anti-GlcCer monoclonal antibody (100 µg/mL) was added and plates were incubated for 1 h at 37 °C, washed three times and then incubated with HRP-conjugated anti-mouse IgG (1:1000 dilution) (Sigma-Aldrich, Germany) for 1 h at 37 °C. After a new washing step, the antigen–antibody complexes were detected with 0.04% orthophenylene-diamine (OPD) in phosphate-citrate buffer at pH 5.0 containing 30 vol. H_2_O_2_ and the absorbance was measured at 490 nm in a spectrophotometer (Bio-Rad, United States).

### 2.6. Immunofluorescence Analysis

Immunofluorescence analysis was performed as described by Xisto et al. [20]. Germinated conidia (incubated for 3, 6 and 18 h), 1 M NaOH-treated and untreated conidia were fixed in 4% paraformaldehyde and adhered on glass slides coated with poly-L-lisine for 10 min. The glass slides with adhered fungal cells were blocked with 3% BSA in PBS for 1 h. Anti-GlcCer was added at 100 µg/mL and incubated at 4 °C overnight. After washing, secondary antibodies conjugated with Alexa Fluor 546 were added and incubated under the same conditions. The slides were washed three times and observed in an Axio Imager 2 fluorescence microscope (Carl Zeiss, Germany).

### 2.7. Germination Assay

*S. aurantiacum* isolates (10^5^ conidia/mL) and anti-GlcCer (100 µg/mL) were added in 24-well plates containing RPMI 1640 and incubated for 3 and 6 h at 37 °C. Afterwards the germinated conidia were counted and the length of the germ tubes was measured using an optical microscope. At least 100 conidia were counted and the germination percentage was calculated as 100 × the ratio of the number of germinated conidia to the total number of cells counted [12,28].

### 2.8. Membrane Stressors’ Susceptibility

*S. aurantiacum* isolates (10^5^ conidia/mL) were incubated in the presence of anti-GlcCer (100 µg/mL) for 3 h at 37 °C in RPMI 1640. Then, Calcofluor white (10 µg/mL) or NaCl (3%) were added to the medium as cell wall and membrane stressors, respectively [16,25]. Control conditions were represented by conidia without stressors or antibody and by conidia with stressor treatment alone. After 24 h incubation, fungal metabolic activity was measured by XTT-reduction assay. Cells growing without anti-GlcCer and both stressors were used as controls.

### 2.9. Filipin Staining of Lipid Raft Domains

Filipin staining was performed according to Rollin-Pinheiro et al. [25]. *S. aurantiacum* WM 06.385 cells (10^5^ conidia/mL) were incubated in the presence of anti-GlcCer (100 µg/mL) for 3 h at 37 °C in RPMI 1640 and fixed with 4% paraformaldehyde. The fixed germinated conidia were stained with 50 µg/mL of filipin diluted in sterile PBS for 2 h at room temperature in the dark. After washing two times with sterile PBS, cells were visualized in an Axio Imager 2 fluorescence microscope (Carl Zeiss, Germany) [25].

### 2.10. Biofilm Formation Assay

*S. aurantiacum* isolates (10^5^ conidia/mL) were added to 96-well polystyrene microplates and incubated in the presence of anti-GlcCer (100 µg/mL) for 24 h at 37 °C in RPMI 1640 (supplemented with 2% glucose and 20% fetal bovine serum), as described by Rollin-Pinheiro et al. [12]. After biofilm formation, a PBS washing step was performed to remove non-adherent cells. Resultant biofilm was quantified using two different methods [12]. Violet crystal was used to assess overall biomass and XTT-reduction assay to measure metabolic activity and, therefore, to evaluate cell viability. Spectrophotometer (Bio-Rad, Hercules, CA, USA) readings at 590 and 490 nm were performed to evaluate biomass and cell viability, respectively.

### 2.11. Adhesion of Germinated Conidia and Biofilm in the Presence of Anti-GlcCer

*S. aurantiacum* cells (10^5^ conidia/mL) were added to 96-well polystyrene microplates and incubated in the presence of anti-GlcCer (100 µg/mL) for 6 and for 24 h at 37 °C in RPMI 1640. At each time point, plates were washed three times with sterile PBS to remove non-adherent cells. For germinated conidia (cells grown for 6 h), adhered cells were counted in five different microscopic fields for each well using an inverted microscope. Adhered biofilms (formed by cells grown for 24 h) were evaluated by comparing the measurements of optical density (660 nm) before and after the removal of non-adherent cells.

### 2.12. Statistical Analysis

All experiments were performed in triplicate, in three independent experimental sets. Statistical analyses were performed using GraphPad Prism version 5.00 for Windows (GraphPad Software, San Diego, CA, USA). One-way analysis of variance using a Kruskal-Wallis nonparametric test was used to compare the differences between groups, and individual comparisons of groups were performed using a Bonferroni posttest. The 90–95% confidence interval was determined in all experiments.

## 3. Results

### 3.1. Structural Analysis of S. aurantiacum Glucosylceramides

**Figure 1:**
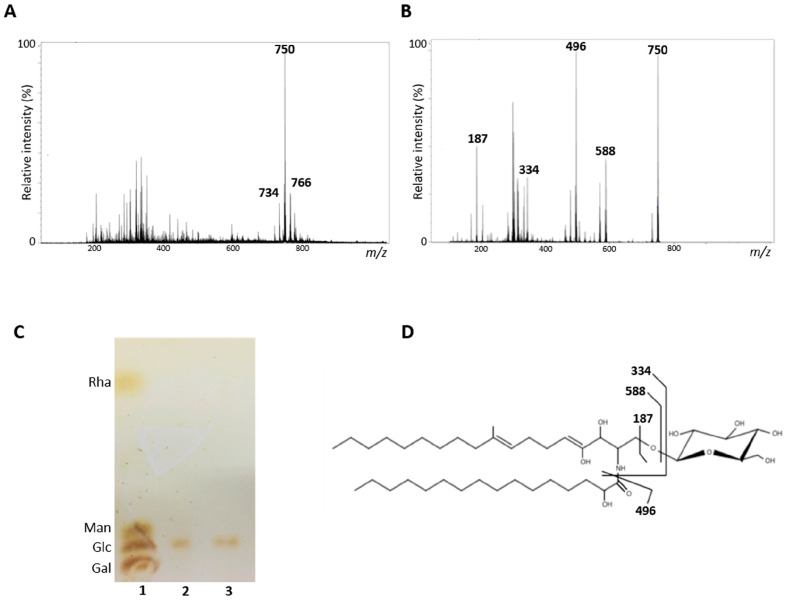
Structural analysis of glucosylceramides (GlcCer) obtained from *S. aurantiacum* isolates. (**A**) Electrospray ionization mass spectrometry ESI-MS (ESI-MS1) of purified GlcCer. (**B**) ESI-MS2 of ion species *m*/*z* 750 observed in ESI-MS1. (**C**) High performance thin layer chromatography of monosaccharides from *S. aurantiacum* GlcCer. 1. Rhamnose (Rha), mannose (Man), glucose (Glc) and galactose (Gal) standards; 2. Hydrolyzed GlcCer of *S. aurantiacum* WM 09.12; 3. Hydrolyzed GlcCer of *S. aurantiacum* WM 06.385. (**D**) Proposed structure for the major GlcCer species found in *S. aurantiacum* WM 09.12 and WM 06.385.

Purified GlcCer obtained from *S. aurantiacum* WM 09.12 and WM 06.385 was analyzed by electrospray ionization mass spectrometry (ESI-MS). GlcCer from both isolates are composed of three molecular ion species: a major lithiated ion species at mass to charge ratio (*m*/*z*) 750 and other two minor species at *m*/*z* 734 and *m*/*z* 766 (Figure 1A). The major ion species at *m*/*z* 750 was submitted to tandem fragmentation (ESI-MS/MS) and three daughter ions were identified at *m*/*z* 588 corresponding to the ceramide mono-lithiated from the parental ion, *m*/*z* 496 corresponding to a hydroxylated C16-fatty acid, and a fragment at *m*/*z* 187 confirmed the presence of a hexose (Figure 1B). These data indicate that the major ion *m*/*z* 750 structure consists of a hexose, a long chain base (9-methyl-4,8-sphingadienine) with an extra hydroxyl group and a C16 fatty acid chain (Figure 1B). After hydrolysis with 3 M TFA, glucose was revealed as the hexose component of GlcCer from both *S. aurantiacum* isolates (Figure 1C). The other two minor species at *m*/*z* 734 and *m*/*z* 766 showed differences in hydroxylation and fatty acid chain lengths.

### 3.2. GlcCer Exposure on S. aurantiacum Surface during Germination

**Figure 2:**
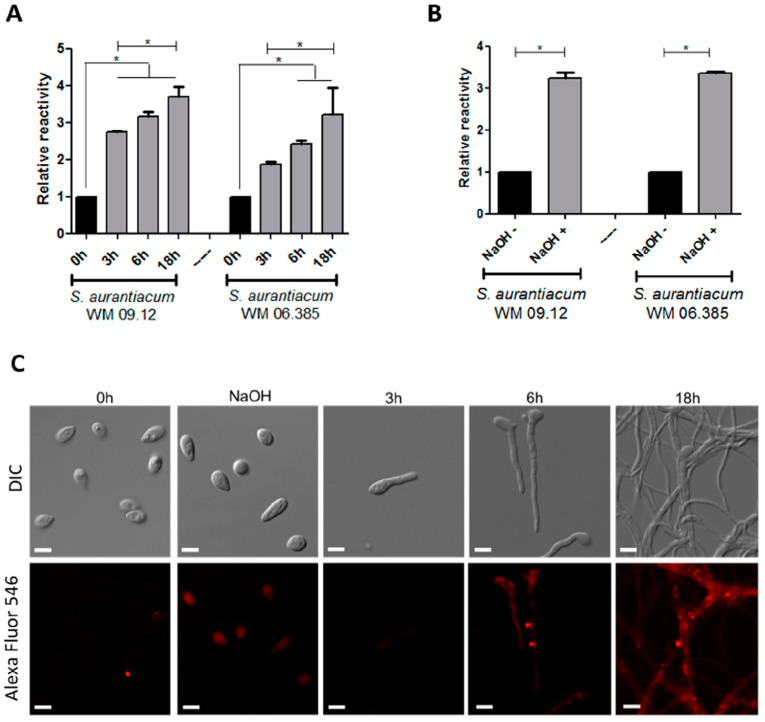
GlcCer exposure on *S. aurantiacum* WM 09.12 and WM 06.385 cell surface detected by monoclonal anti-GlcCer antibody. (**A**) ELISA of anti-GlcCer (100 µg/mL) bound to *S. aurantiacum* cells during different germination time points and (**B**) to conidia after treatment with NaOH 1M. (**C**) Immunofluorescence analysis of anti-GlcCer (100 µg/mL) reactivity to *S. aurantiacum* WM 06.385. Scale bar: 5 µm. * *p* < 0.05.

GlcCer exposure on the *S. aurantiacum* surface was evaluated by the reactivity of anti-GlcCer with fungal cells in different germination stages. Using ELISA, it was observed that anti-GlcCer reactivity increases progressively along germination over time, from 0 h (only conidia) until 18 h when mycelia are already formed (Figure 2A). To evaluate whether melanin and other alkali-soluble molecules present on the surface of conidia could interfere with GlcCer exposure on conidial forms, anti-GlcCer reactivity of intact conidia and NaOH-treated conidia was compared. It was observed that anti-GlcCer reactivity is more than three-fold higher in NaOH-treated conidia (Figure 2B), suggesting that GlcCer is more exposed on the fungal surface when melanin and alkali-soluble molecules are removed.

Immunofluorescence analysis corroborated the ELISA data. Staining with Alexa Fluor 546 was more prominent along the germination process, as well as in NaOH-treated conidia when compared to intact conidia (0 h) (Figure 2C). These results indicate that GlcCer exposure increases during the germination process and that melanin-depleted cells were recognized by anti-GlcCer, confirming that melanin and other alkali-soluble molecules mask the recognition of GlcCer on the surface of *S. aurantiacum* conidia.

### 3.3. Influence of Anti-GlcCer on S. aurantiacum Germination

**Figure 3:**
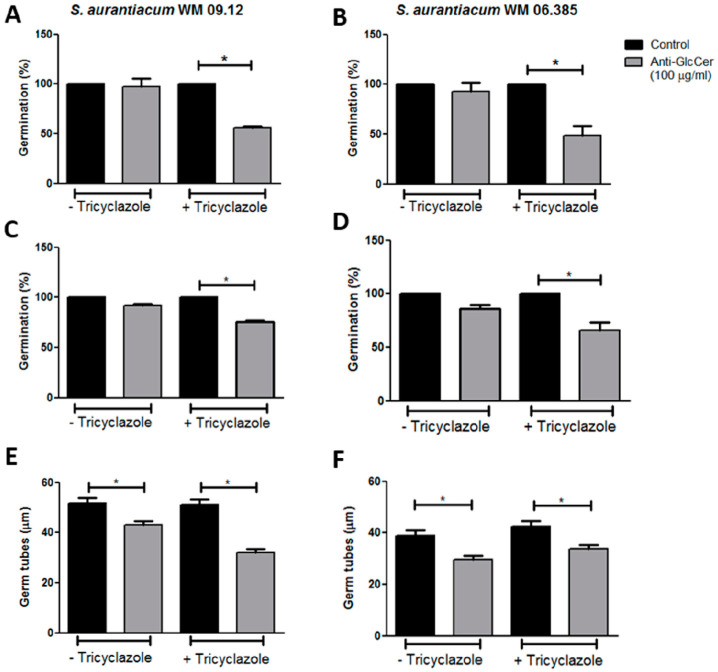
Anti-GlcCer effect in germination of *S. aurantiacum* WM 09.12 (**A**,**C**,**E**) and WM 06.385 (**B**,**D**,**F**). Germination rates of *S. aurantiacum* conidia in the presence of anti-GlcCer (100 µg/mL) for 3 h (**A**,**B**) and 6 h (**C**,**D**). Germ tube lengths were measured after 6 h of germination with anti-GlcCer (**E**,**F**). Conidia were cultivated in the absence (−Tricyclazole) or presence (+Tricyclazole) of tricyclazole (16 µg/mL). * *p* < 0.05.

To study the influence of anti-GlcCer on *S. aurantiacum* germination, intact conidia and conidia grown in the presence of tricyclazole (Dihydroxynaphthalene (DHN)–melanin synthesis inhibitor that does not affect fungal viability) were incubated for 3 and 6 h with 100 µg/mL of anti-GlcCer. Percentage of germination was calculated as 100 x ratio of counts of germinated conidia and total conidia. After 3 h of incubation, anti-GlcCer did not affect intact conidia germination rates but reduced tricyclazole-treated conidia germination in 44.1% and 51.5% for WM 09.12 and WM 06.385 isolates, respectively (Figure 3A,B). After 6 h of incubation, no effect on intact conidia germination was observed, but 24.7% and 34.3% reduction were observed in tricyclazole-treated conidia for WM 09.12 and WM 06.385 isolates, respectively (Figure 3C,D). These data suggest that melanin inhibition is necessary for anti-GlcCer to reduce conidia germination.

In order to better evaluate GlcCer involvement in *S. aurantiacum* growth, germ tube length was measured after each time point of incubation in the presence of anti-GlcCer. No difference in the mean germ tube length was observed after 3 h of incubation for any condition tested in comparison to control. However, after 6 h of incubation, tricyclazole-treated and intact conidia had shorter germ tubes in the presence of anti-GlcCer when compared to control cells (Figure 3E,F). This indicates that not only the number of germinated cells is decreased by anti-GlcCer in the presence of tricyclazole, but also that the length of the germ tubes of both treated and non-treated conidia are affected.

### 3.4. Effect of Anti-GlcCer on Lipid Raft Organization

**Figure 4:**
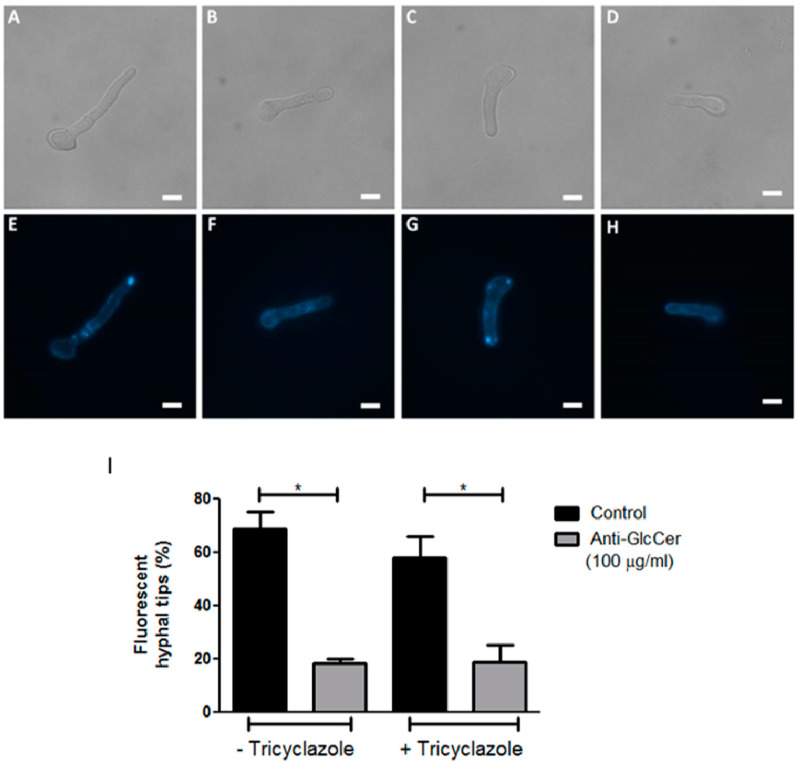
Organization of lipid rafts in *S. aurantiacum* WM 06.385 after 3 h of germination. Filipin staining was performed in untreated (**E**,**F**) and tricyclazole-treated (**G**,**H**) conidia, in the absence (**E**,**G**) or presence of anti-GlcCer (100 µg/mL) (**F**,**H**). In (**A–D**) differential interferential contrast microscopy is presented. Scale bar: 5 µm. The graph in (**I**) represents the quantification of cells that displayed fluorescent hyphal tips. * *p* < 0.05.

As observed in other filamentous fungi, the formation of sterol and sphingolipid-rich membrane domains, called lipid-rafts, seems to be important for hyphal elongation. Since anti-GlcCer decreased *S. aurantiacum* germination, we investigated whether the monoclonal antibody could also affect lipid-raft organization. Intact and tricyclazole-treated conidia were incubated with 100 µg/mL of anti-GlcCer for 3 h, the time point in which a strong reduction of the germination rate was observed. *S. aurantiacum* WM 06.385 was chosen for this analysis because a higher germination inhibition in the presence of anti-GlcCer was detected. After the incubation time, cells were stained with filipin, a polyene macrolide that binds to ergosterol and is commonly used for lipid raft studies. It was observed that control conidia (in the absence of anti-GlcCer) presented lipid-raft accumulation at the apical region of germ tubes, which usually occurs along fungal germination (Figure 4E,G). However, in anti-GlcCer-treated conidia, lipid raft accumulation was not observed and a diffuse stain along the fungal cell surface was found in most cells (Figure 4F,H). It was observed that 68.75% and 58% of untreated and tricyclazole-treated conidia, respectively, presented hyphal tips with intense filipin fluorescence. In contrast, in the presence of anti-GlcCer, 18.30% and 18.75% of untreated and tricyclazole-treated conidia, respectively, presented filipin accumulation in the hyphal tips. These analyses reveal that anti-GlcCer bound to GlcCer on the cell surface leads to lipid-raft disorganization, independently of melanin synthesis inhibition.

### 3.5. S. aurantiacum Susceptibility to Surface Stressors in the Presence of Anti-GlcCer

**Figure 5:**
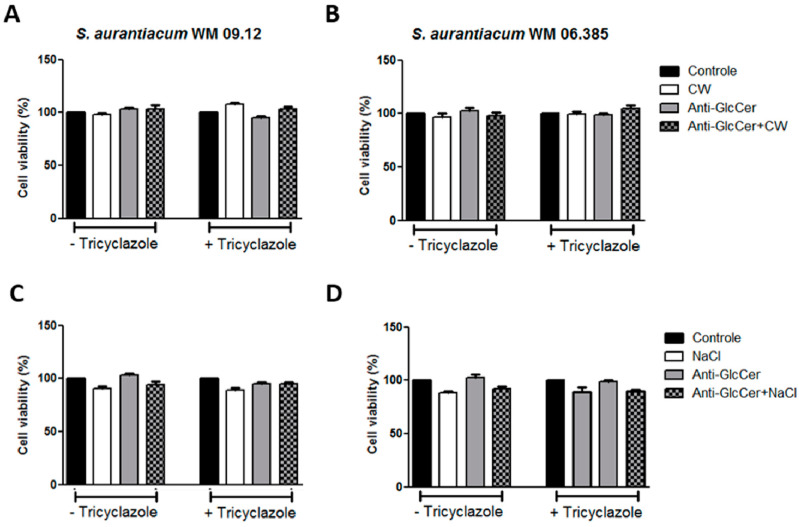
*S. aurantiacum* WM 09.12 (**A**,**C**) and WM 06.385 (**B**,**D**) susceptibility to NaCl and Calcofluor White in the presence of anti-GlcCer (100 µg/mL). *S. aurantiacum* cells were pre-incubated with anti-GlcCer for 3 h and then cell viability was determined by XTT-reduction assay after 24 h incubation with Calcofluor White (10 µg/mL) (**A**,**B**) or NaCl (3%) (**C**,**D**). Conidia were cultivated either with (+) or without (−) tricyclazole (16 µg/mL). CW, Calcofluor White.

Since GlcCer is a surface molecule and anti-GlcCer is able to decrease the germination process and lipid raft organization, we evaluated whether anti-GlcCer is able to affect membrane and cell wall integrity. *S aurantiacum* cells were incubated with anti-GlcCer for 3 h followed by 24 h incubation with Calcofluor White, a cell wall stressor, and NaCl, which causes osmotic stress, both compounds commonly used to investigate the cell wall and plasma membrane integrity. Susceptibility to both stressors was not altered in the presence of anti-GlcCer, even in intact or tricyclazole-treated cells since cell viability was similar to controls and stressors alone (Figure 5). These data indicate that anti-GlcCer does not interfere with the surface integrity.

### 3.6. Influence of Anti-GlcCer on Biofilm Formation

**Figure 6:**
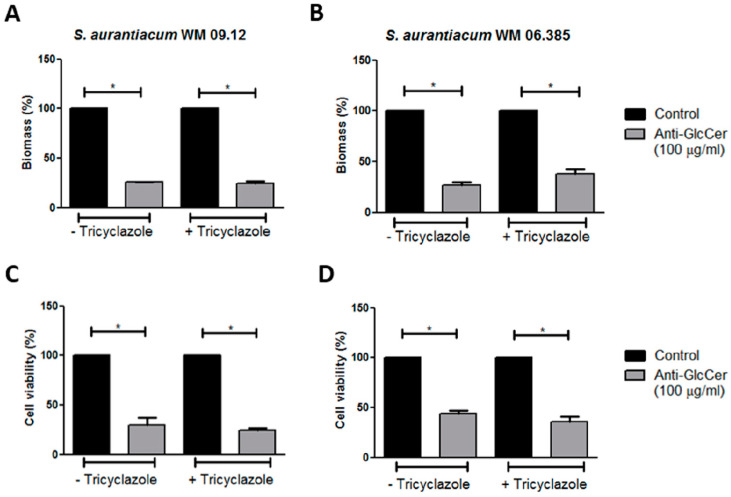
Influence of anti-GlcCer in *S. aurantiacum* WM 09.12 (**A**,**C**) and WM 06.385 (**B**,**D**) biofilm formation. *S. aurantiacum* biofilms formed in the presence of anti-GlcCer (100 µg/mL) for 24 h had total biomass analyzed by crystal violet staining (**A**,**B**) and cell viability determined by XTT-reduction assay (**C**,**D**). Conidia were cultivated in the absence (−) or presence (+) of tricyclazole (16 µg/mL). * *p* < 0.01.

In order to assess the relevance of GlcCer for biofilm formation, *S. aurantiacum* cells were incubated with anti-GlcCer for 24 h at 37 °C. It was observed that growth in the presence of anti-GlcCer reduced biofilm biomass of intact conidia in 74.2% and 73% for *S. aurantiacum* WM 09.12 and WM 06.385, respectively (Figure 6A,B). As for tricyclazole treated conidia, total biomass was reduced in 75.3% and 62% for *S. aurantiacum* WM 09.12 and WM 06.385, respectively (Figure 6A,B). Intact conidia cell viability was reduced in 70.3% and 56% for *S. aurantiacum* WM 09.12 and WM 06.385, respectively (Figure 6C,D). Tricyclazole treated conidia had 75.6% and 64.5% cell viability reduction for *S. aurantiacum* WM 09.12 and WM 06.385, respectively (Figure 6C,D). Intact and tricyclazole-treated cells displayed similar results, indicating that melanization does not affect the effect of anti-GlcCer observed on biofilm formation.

### 3.7. Influence of Anti-GlcCer Adhesion of Germinated Conidia and Biofilm

**Figure 7:**
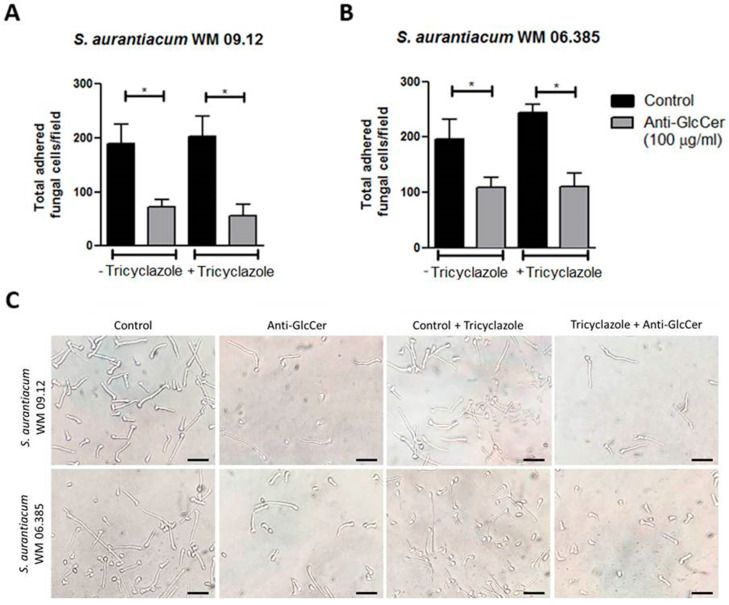
Adhesion of germinated conidia from *S. aurantiacum* to polystyrene after 6 h incubation with anti-GlcCer (100 µg/mL). Total adhered cells of *S. aurantiacum* WM 09.12 (**A**) and WM 06.385 (**B**) were counted in an inverted microscope. (**C**) Representative images of adhered fungal cells to polystyrene. Conidia were cultivated in the absence (−) or presence (+) of tricyclazole (16 µg/mL). * *p* < 0.01. Scale bar: 10 μm.

During biofilm formation assays we observed that fungal growth seemed similar in anti-GlcCer treated conidia and control conditions before the removal of non-adherent cells. Therefore, we hypothesized that the reduced biomass and viability observed in the biofilm formation assay was due to a lesser adherence of anti-GlcCer treated cells to the polystyrene surface. In order to test whether anti-GlcCer affects *S. aurantiacum* adhesion to polystyrene, intact and tricyclazole-treated conidia were incubated for 6 h with anti-GlcCer since it was previously demonstrated that for *Scedosporium* species germinated conidia are the main morphotype found adhered to polystyrene surface. After incubation, the plates were washed to remove non-adherent cells, and then the adhered cells were counted. It was observed that anti-GlcCer-treated cells were significantly less adherent than the control (Figure 7A and/or B). In addition, intact or tricyclazole-treated cells displayed similar results, suggesting that melanin does not interfere with the anti-GlcCer effect on fungal adhesion.

**Figure 8:**
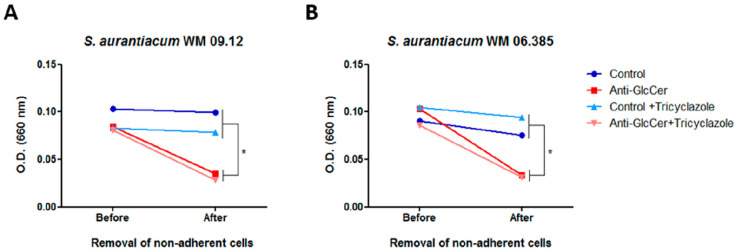
Anti-GlcCer effect in *S. aurantiacum* WM 09.12 (**A**) and WM 06.385 (**B**) biofilm adhesion to polystyrene. Biofilm growth was measured by determining the optical density at 660 nm before and after removal of non-adherent cells. Conidia cultivated in the absence or presence of tricyclazole (16 µg/mL) were used and grown for 24 h in the presence of anti-GlcCer (100 µg/mL). * *p* < 0.05.

To evaluate whether the effect in cell adhesion is extended to biofilm growth, *S. aurantiacum* cells were incubated for 24 h with anti-GlcCer and the optical density (OD) was measured before and after the removal of non-adherent cells. It was observed that after the removal of non-adherent cells, OD was significantly decreased in anti-GlcCer treated conditions, whereas a reduced OD was not observed in controls after the washing step (Figure 8A and/or B), suggesting that anti-GlcCer incubation resulted in less adherent cells that could be removed by the washing step. Intact or tricyclazole-treated cells displayed similar results, suggesting that melanin also does not interfere with the effect of anti-GlcCer on biofilm adhesion. These data indicate that GlcCer is relevant for *S. aurantiacum* adhesion to polystyrene, an essential step for biofilm formation, and that anti-GlcCer does not only affect fungal growth, but also the adhesion to polystyrene surface.

## 4. Discussion

Cell components can help to elucidate fungal biological processes, with the cell wall being a structure highlighted by its importance for morphogenesis and interaction with host cells [30]. GlcCer is the main neutral glycosphingolipid present in fungal cell wall and membrane and has a unique chemical structure compared to GlcCer in mammals and plants, indicating that this molecule may be a potential target for antifungal drugs [31]. In fungi, GlcCer has a conserved structure, with minor differences observed in different morphotypes, isolates and species [15]. GlcCer synthesis is performed by the action of a variety of enzymes, which includes a reductase to form 3-ketodihydrosphingosine, a ceramide synthase that adds a fatty acid chain to the sphingoid base, two sequential desaturases that add double bonds at carbons 4 and 8, a methyl transferase which adds the methyl group at carbon 9 and, finally, the GlcCer synthase that brings the sugar unit to the molecule [32]. Studies revealed that Δ8-desaturation and C9-methylation found in fungal GlcCer are not present in mammalian ones, suggesting that genes encoding those desaturase and methyl transferase are absent in mammals [31]. Although GlcCer is considered a conserved molecule in fungi, they are able to produce structures with different backbones, which highlights the complexity of fungal sphingolipids. However, genetic mechanisms of the biosynthetic pathway of fungal sphingolipids are still poorly understood and more studies are needed to clarify its dynamics.

Structural analysis of GlcCer from both *S. aurantiacum* isolates used in this work identified a major molecular ion at *m*/*z* 750 that contains glucose, 9-methyl-4,8-sphingadienine with an extra hydroxyl group and 2-hydroxy-hexadecanoic acid. *Lomentospora prolificans*, *Rhizopus stolonifer*, *R. microspores* and sclerotic cells of *Fonsecaea pedrosoi* also showed the presence of this ion at *m*/*z* 750 [20,33,34]. Two minor species were also found in both *S. aurantiacum* isolates at *m*/*z* 734 and *m*/*z* 766. The ion at *m*/*z* 734 is the major glucosylceramide present in most *Scedosporium* species analyzed such as *S. apiospermum*, *S. boydii*, *S. ellipsoideum*, *S. angustum*, as well as another *S. aurantiacum* isolate [18,19,28]. The ion at *m*/*z* 766 is also present as a minor species in *S. apiospermum*, *R. stolonifer* and *R. microspores* [28,34]. The molecular ion observed at *m*/*z* 766 corresponds to a GlcCer with a ceramide containing a C20 long chain base instead of the C18 long chain base present in both *m*/*z* 750 and *m*/*z* 734 species [18,20,34]. Regarding the monosaccharide components, it is known that fungal sphingolipids usually contains glucose or galactose as sugar unit, although the impact of this variation on sphingolipid function has not yet been demonstrated. Whereas some fungi, such as *Sporothrix schenkii* and *Aspergillus* species possess both glucose and galactose, in the majority of pathogenic fungi studied so far, such as *Fusarium* species, *Paracoccidioides brasiliensis*, *Cryptococcus neoformans* and *Candida albicans*, only glucose is detected [14,35]. Our results revealed that glucose is found in *S. aurantiacum* whereas other monosaccharides such as galactose, mannose and rhamnose were absent, which corroborates with other studies that also demonstrate the presence of glucose in other Scedosporium species, such as *S. apiospermum*, *S. boydii*, *S. ellipsoideum* and *S. angustum* [18,28].

Monoclonal anti-GlcCer antibodies have been used to detect the presence of GlcCer on conidial and mycelial forms of different *Scedosporium* species, such as *S. boydii*, *S. apiospermum*, *S. minutisporum* and other *S. aurantiacum* isolates [18,19,28]. However, little is known about the role of GlcCer during fungal germination. We decided to observe the expression of GlcCer during the germination process using anti-GlcCer antibody. An increase in antibody reactivity was detected during either the germination, since *S. aurantiacum* has hyaline hyphae, or after treatment of conidia with NaOH, which removes melanin and other alkali molecules present in the conidia cell surface, facilitating the recognition of GlcCer by the anti-GlcCer antibody on both *S. aurantiacum* isolates surface. Similar results were also found for *L. prolificans* and *F. pedrosoi* [20,33]. It has already been shown that the cell wall of several pathogenic fungi present alkali-soluble molecules, which include α-glucans, mannans and, in the case of *Scedosporium* species, rhamnomannans, peptidorhamomannans and some pigments, such as melanin [36,37]. Regarding melanin, it is present on conidia cell wall, masking some antigenic molecules, such as GlcCer, but not on hyphal structures of hyaline fungi, which justifies why anti-GlcCer antibodies better recognize S. aurantiacum hyphae or NaOH-treated conidia.

GlcCer are important molecules for growth, differentiation and pathogenesis of different fungal species. Since no mutant strain for GlcCer biosynthesis pathway has been developed for *Scedosporium* species so far, synthetic inhibitors and monoclonal anti-GlcCer antibodies have become important tools for GlcCer functional studies. Anti-GlcCer protects mice against invasive cryptococcosis as well as reducing *Colletotrichum gloeosporioides*, *S. apiospermum* and *S. minutisporum* germination [19,26,27]. Our results showed that *S. aurantiacum* conidia germination rates were not affected by the presence of anti-GlcCer. However, monoclonal antibody reduced germination rates of tricyclazole-treated conidia, probably due to an increase in GlcCer exposure as a consequence of melanin biosynthesis inhibition. In addition, anti-GlcCer led to shorter germ tubes in germinated conidia, both treated with tricyclazole or not. Collectively, these results indicate that GlcCer is important for the *S. aurantiacum* germination process.

Several studies reported the effect of anti-GlcCer in fungal morphological transition, but the mechanisms remain unknown. *Aspergillus nidulans* mutant strains lacking sphingolipid biosynthesis genes were not capable of promoting polarized growth and did not present lipid rafts accumulation in the hyphal tips [16,38]. In this context, we investigated with the use of filipin stain if anti-GlcCer could affect lipid raft organization. Our results showed that, in the presence of anti-GlcCer, germinated cells did not show filipin accumulation in the hyphal tips as compared to control cells. These data indicate that binding of anti-GlcCer to GlcCer molecules on the cell surface disrupts the organization of lipid raft domains, an important step for polarized growth in filamentous fungi, which corroborates the germination results, in which anti-GlcCer affects hyphae elongation.

Since GlcCer seems to play a major role in germination and lipid raft organization of *S. aurantiacum*, we investigated if anti-GlcCer could also interfere in cell surface integrity. It was observed that, in the presence of anti-GlcCer, susceptibility to membrane stressors remained unchanged when compared to control cells. We hypothesize that the binding of the monoclonal antibody to GlcCer molecules does not interfere with GlcCer synthesis, or that the fungus could trigger a compensatory mechanism after the loss of surface integrity. However, more studies are needed to elucidate the effect of anti-GlcCer on membrane integrity.

Biofilm is an important characteristic that can enhance fungal virulence, protection against host immune response and resistance to antifungal drugs [39]. *Scedosporium* species form biofilm on different surfaces, such as polystyrene, glass, central venous catheter and lung epithelial cells [11,12]. It was observed that *S. aurantiacum* produces a robust biofilm after 24 h of incubation, whereas *S. boydii* only displayed the same biofilm aspects after 48 h [12]. Previous results demonstrated that myriocin reduced biofilm formation of *A. fumigatus* and *S. boydii*, showing the relevance of sphingolipids in this growth process [24,25]. In the present work it was seen that, in the presence of anti-GlcCer, *S. aurantiacum* germinated conidia and biofilms could not adhere to the polystyrene surface with the same efficacy as control cells. A decrease in biomass and cell viability of *S. aurantiacum* biofilms grown in the presence of anti-GlcCer was demonstrated in this work, suggesting that GlcCer is relevant for adhesion of fungal cells in an abiotic surface, an essential step for biofilm formation. Tricyclazole did not influence *S. aurantiacum* adhesion and biofilm formation, suggesting that melanin inhibition affects conidia germination, especially by exposing GlcCer to the action of anti-GlcCer antibodies that reduces the germination speed, but no effect is observed on fungal adhesion to abiotic surfaces.

In summary, anti-GlcCer monoclonal antibodies are a useful tool to study GlcCer functions on *S. aurantiacum* cells. GlcCer characterized in this work plays an important role in germination, lipid raft organization, fungal cell adhesion and, consequently, biofilm establishment of *S. aurantiacum* isolates. The presented data contribute to the elucidation of the functions and relevance of the surface molecule in *S. aurantiacum* during growth processes. Since GlcCer is important for fungal growth and a potential target for new antifungal therapies, more studies are needed for a better understanding of the role of this molecule in fungal pathogenesis and interaction with host cells.
